# Supplementary material for: Two (or more) for one: Identifying classes of household energy- and water-saving measures to understand the potential for positive spillover
Source: PLoS One. 2022 Jul 5;17(7):e0268879. doi: 10.1371/journal.pone.0268879 (PMC9255758; doi:10.1371/journal.pone.0268879)
Supplement: S1 File — (PDF) [file pone.0268879.s001.pdf]

Introduction and Consent

Welcome!

In this survey, you will be asked about **actions you take that save water and energy in your home**.

It will take approximately 10 minutes.

At the end of the survey you will be asked to enter your email in order to receive your $20 gift card.

This research has been approved by the Institutional Review Board of University of California, Davis. You may discontinue participation at any time. Your responses will be confidential; no identifying information, such as your name or address, will be accessible outside the research team, used in analysis, or retained beyond February 1, 2018. Only one gift card will be awarded per participating household. Please complete the survey by December 20, 2018. Gift cards will be sent via email by December 22. If you have questions or concerns please contact the lead researcher, Dr. Angela Sanguinetti at asanguinetti@ucdavis.edu. By proceeding you confirm that you were the original recipient of the survey invitation and consent to the conditions outlined above.

Actions

Which of the following actions do you regularly take at home?
Mark all that apply.

- [ ] Check for thermal leaks (e.g., around doors, windows, or baseboards)
- [ ] Use a broom instead of hose when cleaning driveways, walkways, decks, or patios
- [ ] Turn air conditioner down / off at night in the summer
- [ ] Clean and/or replace air conditioner filters
- [ ] Turn off lights when leaving room
- [ ] Set water heater temperature to 120 degrees
- [ ] Use a cloth instead of hose when cleaning lawn furniture and outdoor toys / sports equipment
- [ ] Hang / air dry laundry
- [ ] Caulk or seal around leaky doors, window frames, or baseboards
- [ ] Check for showerhead, faucet, or toilet leaks

- ☐ Turn heater down / off at night in the winter
- ☐ Fully load clothes washer
- ☐ Turn off TV when not in use
- ☐ Clean light bulbs
- ☐ Turn off computer when not in use
- ☐ None of the above

Which of the following actions do you regularly take while bathing / grooming?

Mark all that apply.

- ☐ Turn off water while shaving
- ☐ Capture cold water in a bucket while waiting for hot water
- ☐ Turn off water while scrubbing face / hair / body
- ☐ Take short showers (5 minutes or less)
- ☐ Reuse bath towels
- ☐ Turn off water while brushing teeth
- ☐ Turn off water while soaping hands
- ☐ None of the above

Which of the following actions do you regularly take in the kitchen?

Mark all that apply.

- ☐ Fully load dishwasher
- ☐ Cover pots and pans when cooking
- ☐ Reuse cooking water after boiling (e.g., to make soup or water plants)
- ☐ Close refrigerator door quickly after opening
- ☐ Turn off water when scrubbing fruits and vegetables
- ☐ Clean refrigerator coils
- ☐ Turn off water when scraping / scrubbing dishes
- ☐ None of the above

Do you have a yard?

- ○ Yes
- ○ No

Which of the following do you have at your home?
Mark all that apply.

- ☐ Dishwasher
- ☐ Pool
- ☐ Spa
- ☐ None of the above

Which of the following actions do you regularly take in your yard?
Mark all that apply.

- ☐ Check for irrigation system / sprinkler / hose leaks
- ☐ Water different plants according to their different needs
- ☐ Compost grass / leaves / food scraps for soil nutrients
- ☐ Mulch leaves and leave in yard rather than disposing of them
- ☐ Check soil moisture level before watering
- ☐ Use multiple irrigation / watering start times
- ☐ Ensure water isn't running onto paved surfaces during watering
- ☐ Stop watering when it rains
- ☐ Adjust irrigation / sprinkler timer monthly
- ☐ Water only at dawn or dusk
- ☐ Trim plants around sprinkler heads
- ☐ None of the above
- ☐ My yard does not require watering
- ☐ Someone else in my household does the yard work

Which of the following actions do you regularly take for your pool / spa?
Mark all that apply.

- ☐ Reduce or shorten pool pump cycles
- ☐ Cover pool / spa when not in use
- ☐ Check for pool / spa leaks
- ☐ None of the above

Here are the energy-saving and water-saving actions you regularly take at home:

Around the house:

${q://QID62/ChoiceGroup/SelectedChoices}

Bathing / grooming:
${q://QID59/ChoiceGroup/SelectedChoices}

In the kitchen:
${q://QID74/ChoiceGroup/SelectedChoices}

In the yard:
${q://QID67/ChoiceGroup/SelectedChoices}

Pool / spa:
${q://QID68/ChoiceGroup/SelectedChoices}

Which of the following describe your reasons for taking these actions?
Mark all that apply.

- [ ] Pressure from other member(s) of my household
- [ ] To care for the environment
- [ ] I feel guilty if I am wasteful
- [ ] To be efficient / save money
- [ ] Other

Investments

Which of following energy-saving or water-saving investments / measures do you have in your home?
Mark all that apply.

- [ ] Smart thermostat
- [ ] Weather-stripping on doors or windows
- [ ] Water pressure regulator valves
- [ ] Water displacement device in toilet(s)
- [ ] High-efficiency or double-paned windows
- [ ] Insulation around hot water pipes
- [ ] ENERGY STAR computer
- [ ] ENERGY STAR dryer

- ☐ ENERGY STAR TV
- ☐ Low-flow faucet aerator(s)
- ☐ Tankless water heater
- ☐ Whole-house fan
- ☐ High-efficiency toilets
- ☐ Clothes dryer with moisture sensor
- ☐ High-efficiency showerhead(s)
- ☐ ENERGY STAR refrigerator
- ☐ Motion sensors, dimmers, or timers for lights
- ☐ Insulation around hot water tank
- ☐ Hot water recirculation pump
- ☐ LED lights
- ☐ Insulation in walls, ceilings, roofs, attic
- ☐ None of the above

Were any of these investments made **in the past 12 months**?

	Yes	No	Not sure
» High-efficiency showerhead(s)	○	○	○
» Low-flow faucet aerator(s)	○	○	○
» Insulation around hot water pipes	○	○	○
» Insulation around hot water tank	○	○	○
» Tankless water heater	○	○	○
» High-efficiency toilets	○	○	○
» Water displacement device in toilet(s)	○	○	○
» Hot water recirculation pump	○	○	○
» Water pressure regulator valves	○	○	○
» LED lights	○	○	○
» Insulation in walls, ceilings, roofs, attic	○	○	○
» High-efficiency or double-paned windows	○	○	○
» Weather-stripping on doors or windows	○	○	○
» ENERGY STAR refrigerator	○	○	○
» Clothes dryer with moisture sensor	○	○	○
» Smart thermostat	○	○	○
» Motion sensors, dimmers, or timers for lights	○	○	○
» Whole-house fan	○	○	○
» ENERGY STAR dryer	○	○	○

	Yes	No	Not sure
» ENERGY STAR TV	◯	◯	◯
» ENERGY STAR computer	◯	◯	◯
» None of the above	◯	◯	◯

Which of following energy-saving or water-saving investments / measures do you have in your yard?

Mark all that apply.

- ☐ Replaced lawn with artificial turf
- ☐ Drip irrigation
- ☐ Replaced high-water use plants with low water-use plants
- ☐ Pool or spa cover
- ☐ Permeable pavement
- ☐ Soil moisture sensor system
- ☐ Weather-based irrigation controller
- ☐ Put mulch at the base of trees / bushes / shrubs
- ☐ Solar-powered garden lights
- ☐ Changed grass to native plants
- ☐ Rotating sprinkler heads
- ☐ Mulching lawnmower
- ☐ Rainwater catchment system
- ☐ Hose faucet timer
- ☐ Graywater system
- ☐ None of the above

Were any of these investments made **in the past 12 months**?

	Yes	No	Not sure
» Weather-based irrigation controller	◯	◯	◯
» Rotating sprinkler heads	◯	◯	◯
» Soil moisture sensor system	◯	◯	◯
» Hose faucet timer	◯	◯	◯
» Rainwater catchment system	◯	◯	◯
» Drip irrigation	◯	◯	◯
» Permeable pavement	◯	◯	◯
» Mulching lawnmower	◯	◯	◯

	Yes	No	Not sure
» Changed grass to native plants	◯	◯	◯
» Replaced high-water use plants with low water-use plants	◯	◯	◯
» Put mulch at the base of trees / bushes / shrubs	◯	◯	◯
» Replaced lawn with artificial turf	◯	◯	◯
» Graywater system	◯	◯	◯
» Pool or spa cover	◯	◯	◯
» Solar-powered garden lights	◯	◯	◯
» None of the above	◯	◯	◯

Here are the energy-saving and water-saving measures you have invested in:

For your home:
${q://QID99/ChoiceGroup/SelectedChoices}

For your yard:
${q://QID101/ChoiceGroup/SelectedChoices}

Which of the following describe your reasons for taking these actions?
Mark all that apply.

- ☐ I received a rebate
- ☐ Someone else in my household made the decision / purchase
- ☐ To be efficient / save money in the long term
- ☐ To care for the environment
- ☐ Other ______

Attitudes

How much do you agree or disagree with the following statements?

	Strongly agree	Somewhat agree	Neither agree nor disagree	Somewhat disagree	Strongly disagree
I carefully examine my household **energy bills**	◯	◯	◯	◯	◯
I have put a lot of effort into **saving energy** at home	◯	◯	◯	◯	◯

	Strongly agree	Somewhat agree	Neither agree nor disagree	Somewhat disagree	Strongly disagree
I wish I knew more about how to **save energy** at home	◯	◯	◯	◯	◯

How much do you agree or disagree with the following statements?

	Strongly agree	Somewhat agree	Neither agree nor disagree	Somewhat disagree	Strongly disagree
I carefully examine my household **water bills**	◯	◯	◯	◯	◯
I have put a lot of effort into **saving water** at home	◯	◯	◯	◯	◯
I wish I knew more about how to **save water** at home	◯	◯	◯	◯	◯

Demographics

What is your gender?

◯ Male

◯ Female

◯ Other

What is your age?

[⌄]

What is the highest level of school you have completed?

◯ Less than high school

◯ High school diploma or GED

◯ Some college but no degree

◯ Associate's degree

◯ Bachelor's degree

◯ Master's degree

◯ Doctoral degree

Household Characteristics

Do you own or rent your home?

- ◯ My home is owned by me and/or someone else in my household
- ◯ My home is rented
- ◯ Other

How many people typically live in your home?

- ◯ 1
- ◯ 2
- ◯ 3
- ◯ 4
- ◯ 5
- ◯ 6
- ◯ 7
- ◯ 8 or more

Who lives in your home with you? (Select all that apply)

- ☐ My spouse/partner
- ☐ My child/children under 18
- ☐ My adult child/children
- ☐ Other adult relative(s)
- ☐ Non-related roommates
- ☐ Pet(s)
- ☐ Other

Who pays your household water and energy bills? (Select all that apply)

- ☐ I do
- ☐ My spouse/partner
- ☐ Other

What is your annual household income?

[▾]

Experience with Reports

Do you recall getting personalized Home Water Reports by mail or email in the past year?

- ◯ Yes
- ◯ No
- ◯ Not sure

Did you look at these reports?

- ◯ Yes, most of them
- ◯ Yes, once or twice
- ◯ No
- ◯ Not sure

Did the reports help you take actions to save water at home?

- ◯ Yes
- ◯ No
- ◯ Not sure

Did the reports help you save money on your water bills?

- ◯ Yes
- ◯ No
- ◯ Not sure

Identifying Information

Please enter your **home address** so we can verify you are the original recipient of our survey invitation:
We will not use your mailing address for any other purpose.

Street number and name: __________

City, state and ZIP code: __________

Please enter your **email address** so we can send you your $20 Starbucks e-gift card:
We will not use your email address for any other purpose. Your gift card will be sent on or before December 1st.

Thank you very much for participating!

Please use the space below to share anything else you would like us to know about your household water and energy use:

Survey Powered By Qualtrics
