# Supplementary material for: Two (or more) for one: Identifying classes of household energy- and water-saving measures to understand the potential for positive spillover
Source: PLoS One. 2022 Jul 5;17(7):e0268879. doi: 10.1371/journal.pone.0268879 (PMC9255758; doi:10.1371/journal.pone.0268879)
Supplement: S2 File — (DOCX) [file pone.0268879.s002.docx]

Variable Key / Answer Codes:

Age

- 1 = 18
- 2 = 19
- ...
- 98 = 115

Gender

- 1 = male
- 2 = female
- 3 = other

School (education)

- 1 = less than high school
- 2 = high school / GED
- 3 = some college, no degree
- 4 = associates
- 5 = bachelors
- 6 = masters
- 7 = phd

Income -

- 1 - less than 10,000
- 2 - 10,000 - 19,999
- 3 - 20,000 - 29,999
- .....
- 11 - 100,000 - 149,999
- 12 - 150,000 - 199,000
- ...
- 15 = more than 300,000

ownRent

- 1 = own
- 2 = rent
- 3 = other

sizeHome - how many people

- 1 - 1
- 2 - 2
- ....
- 8 = 8 or more

paysBillsM - who pays your household bills (select all that apply) - I do

- 1 = yes (checked)
- 0 = no (not checked)

examineEnergy(water) --> I carefully examine my household energy bills

- 11(16) = strongly agree
- 12(17) = somewhat agree
- 13(18) = neither agree nor disagree
- 14(19) = somewhat disagree
- 15(20) = strongly disagree

effortEnergy(water) --> I have put a lot of effort into saving water at home

- 11(16) = strongly agree
- 12(17) = somewhat agree
- 13(18) = neither agree nor disagree
- 14(19) = somewhat disagree
- 15(20) = strongly disagree

knewMoreEnergy (water) --> I wish I knew more about how to save water at home

- 11(16) = strongly agree
- 12(17) = somewhat agree
- 13(18) = neither agree nor disagree
- 14(19) = somewhat disagree
- 15(20) = strongly disagree

Reasons for taking actions (investments) - someoneElse, beEfficientInv, careEnvInvest

- 1 = yes (checked)
- 0 = no (not checked)

Reasons for taking actions (measures) - pressureOther, beEfficientSave, feelGuilty, careEnviro

- 1 = yes (checked)
- 0 = no (not checked)

The remainder of the variables represent energy- and water-efficient measures and can be easily identified because their labels are an abridged version of the full measure names spelled out in the manuscript.

Empty cells mean the participant skipped the question.
